# Neonatal resuscitation for bradycardia (HR < 60 bpm)—an alternate approach using an ovine model

**DOI:** 10.1038/s41390-025-04444-9

**Published:** 2025-10-07

**Authors:** Mausma Bawa, Sylvia Gugino, Justin Helman, Nicole Bradley, Lori Nielsen, Arun Prasath, Clariss Blanco, Munmun Rawat, Praveen Chandrasekharan

**Affiliations:** 1 Division of Neonatology, Department of Pediatrics, University at Buffalo, Buffalo, NY USA; 2 Department of Pediatrics, Boston Children’s Hospital, Harvard Medical School, Boston, MA USA; 3 Division of Neonatology, Department of Pediatrics, UT Southwestern, Dallas, TX USA

## Abstract

**Background:**

The International Liaison Committee on Resuscitation recommends initiating chest compressions (CC) in newborns when heart rate (HR) is <60 beats per minute (bpm) after 30 s of effective positive pressure ventilation (PPV).

**Methods:**

Near-term lambs with asphyxia induced bradycardia (HR < 60 bpm) were randomized to: (i) control (*n* = 6)— Resuscitation per current Neonatal Resuscitation Program (NRP) (ii) study (*n* = 6)—PPV continued until HR = 0, only then CC with PPV were administered in a ratio of 3:1 until return of spontaneous circulation (ROSC). Outcomes included timing, incidence of ROSC, CC requirement, blood gases, and peak coronary, carotid, and pulmonary blood flow.

**Results:**

The time to achieve ROSC was not different between groups (3 ± 2 min vs. 1.6 ± 1 min in study (*p* = 0.17). Only 1/6 lambs in study group required CC versus 6/6 in control group (*p* = 0.015). At ROSC, the study group had significantly lower arterial PaCO2 (47 ± 5 mmHg vs. 94 ± 18 mmHg, *p* < 0.01) and higher arterial PaO_2_ (148 ± 53 mmHg vs. 54 ± 12 mmHg, *p* < 0.01). The asynchronous external CC in the control group contributed to the loss of inherent cardiac activity.

**Conclusion:**

Prioritizing ventilation during bradycardia reduced need for CC, facilitated faster ROSC, and improved gas exchange in an ovine model.

**Impact:**

- Prioritizing ventilation over chest compressions beyond 30 s for bradycardia during neonatal resuscitation improves outcomes, reducing the need for chest compressions and accelerating the time to return of spontaneous circulation.
- Pulseless electrical activity occurs before complete cardiac arrest.
- Focusing on initiating chest compressions at a specific heart rate will distract providers from prioritizing ventilation.

## Introduction

The International Liaison Committee on Resuscitation (ILCOR) and the American Academy of Pediatrics Neonatal Resuscitation Program (AAP-NRP) recommend initiating chest compressions (CC) if an infant’s heart rate (HR) remains less than 60 beats per minute (bpm) after 30 s of effective ventilation.^1,2^ During asphyxia, the stages of cardiac disturbances before cardiac arrest typically progress from initial sinus tachycardia to bradycardia and potential arrhythmias like ventricular tachycardia, then transition to pulseless electrical activity (PEA) and finally culminate in asystole.^3^ Currently, there is no direct clinical evidence supporting the initiation of CC during neonatal bradycardia (HR < 60 bpm). According to ILCOR, using an electrocardiogram (EKG) for HR assessment in the delivery room may increase the number of infants receiving CC. Specifically, an observational study suggests that 35 more live-born infants per 1000 (ranging from 1 fewer to 113 more) may receive CC when EKG is used compared to traditional methods like auscultation and pulse oximetry.^2,4^ Left ventricular filling and coronary blood flow occur during myocardial relaxation (diastole).^5^ Asynchronously compressing the chest 90 times a minute could interfere with the inherent heartbeat (bradycardia) and impair left ventricular filling and myocardial perfusion in the presence of a patent ductus.^6^

Previously, a study involving pediatric patients showed that chest compressions in bradycardic patients with poor perfusion improved survival at discharge from the hospital.^7^ However, in a recent large study involving 5592 pediatric patients with invasive access to measure jugular venous pressure in the pediatric intensive care unit, 50% had bradycardia with poor perfusion at the initiation of CC, and nearly one-third of these patients progressed to pulselessness in-hospital cardiac arrest despite resuscitation.^8^ Survival was significantly lower for children who progressed to pulselessness despite resuscitation compared to those who were initially pulseless.^8^ Asynchronous CC can hamper ventricular filling. In the presence of a patent ductus arteriosus (PDA), ductal steal may occur, further compromising systemic and myocardial perfusion and worsening this situation. Without adequate clinical evidence and follow-up data, it remains unknown if initiating CC in neonatal bradycardia is beneficial or harmful.

We hypothesized that avoiding CC and prioritizing positive pressure ventilation (PPV) along with epinephrine (EPI) if needed during neonatal resuscitation in a near-term asphyxia-induced bradycardia model may establish adequate cerebral and coronary perfusion, leading to a higher incidence and shorter time to return of spontaneous circulation (ROSC). Our objectives were to evaluate: (i) the timing and incidence of ROSC, (ii) the frequency of CC administration, and (iii) gas exchange and hemodynamic parameters.

## Methods

### Instrumentation

The Institutional Animal Care and Use Committee (IACUC# PED10085N) approved the study protocol at the State University of New York at Buffalo. All experiments were conducted in accordance with animal ethical guidelines and in compliance with the ARRIVE guidelines (see supplemental document). Near-term, time dated pregnant ewes (Polypay breed) at 138–140 days of gestation were used in this study. Following an overnight fast, ewes were administered intravenous diazepam and ketamine and were intubated with a 10.0 mm cuffed endotracheal tube (ETT). Ventilation was provided with 100% O_2_, and general anesthesia was maintained using 2–3% inhaled isoflurane, as previously described.^9^ Ewes were continuously monitored with a pulse oximeter and an end-tidal CO_2_ (ETCO_2_) monitor. After performing a cesarean section, the fetal lamb was partially exteriorized and intubated with a 4.5 mm cuffed ETT. Fetal lung fluid was drained passively by gravity, and the ETT was occluded to prevent the entry of air. A 2 mm left carotid arterial flow probe (Transonic Systems, Ithaca, New York) was placed to measure cerebral blood flow. A catheter was placed in the brachial artery with the tip directed towards the aortic arch for invasive arterial blood pressure monitoring and to collect blood samples. The right jugular vein was catheterized for blood draws and fluid/medication administration. A left thoracotomy incision was made in the chest at the fourth intercostal space, and forceps were used to spread the intercostal muscle. A small window in the pericardium was carefully excised, and a 2 mm flow probe was placed around the left circumflex coronary artery under the left atrial appendage. The left pulmonary artery (LPA) was accessed, and a 4–6 mm flow probe was placed to measure blood flow. The chest wall was closed in layers. A pulse oximeter (NONIN Equanox, Nonin Medical Inc., Plymouth, MN) was placed on the right forelimb for continuous SpO_2_ monitoring, and a three-lead EKG (ECG100C, Goleta, CA) was applied to the lamb. Lambs were humanely euthanized with a dose of intravenous pentobarbitone (Fatal-Plus, Vortech Pharmaceuticals, Dearborn, MI) at the conclusion of the experimental protocol.

### Induction of bradycardia

Asphyxia was induced by umbilical cord occlusion until the HR was 60 bpm or the diastolic blood pressure (DBP) ≤ 10 mmHg. Based on our previous study,^10^ even though low diastolic blood pressures were adequate to achieve ROSC, we added the DBP criteria as lambs below this pressure and higher HR often have PEA. Lambs were excluded if there was electrical activity on the EKG, and no blood flow in the real time waveforms on the BIOPAC snapshot. The lambs were then randomized either to the (i) Control group or the (ii) Study group.

**Fig. 1:**
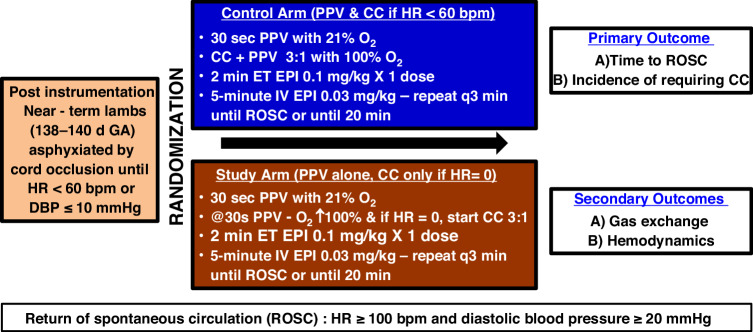
Following instrumentation, near-term lambs of 138–140 days gestation were asphyxiated by umbilical cord occlusion until the heart rate (HR) was <60 bpm or diastolic blood pressure (DBP) was ≤10 mm Hg. The lambs were then randomized into either the control group (resuscitation following current NRP guidelines) or the study group. Positive pressure ventilation (PPV) was initiated with 21% oxygen. After 30 s of ventilation, oxygen was titrated up to 100%, and PPV was continued. Chest compressions (CC) were initiated in the study group only if bradycardia progressed to full arrest (HR = 0). Epinephrine (EPI) was administered in both groups according to NRP guidelines. Resuscitation continued until return of spontaneous circulation (ROSC) or for a maximum of 20 min. ROSC was defined as HR > 100 bpm and DBP ≥ 20 mmHg. The timing and incidence of ROSC, the need for CC, blood gas parameters, peak coronary blood flow (CoBF), peak left carotid blood flow (CaBF), and peak left pulmonary blood flow (PBF) were recorded. HR heart rate, DBP diastolic blood pressure, PPV positive pressure ventilation, CC chest compressions, ET endotracheal, EPI epinephrine, IV intravenous, ROSC return of spontaneous circulation.

#### Resuscitation protocol (Fig. 1)

##### Control group

Following asphyxia, PPV was initiated as per the current NRP recommendations with 21% oxygen. After 30 s of effective ventilation, if the HR remained <60 bpm, supplemental oxygen was increased to 100%, and CC were initiated with a 3:1 compression-to-ventilation ratio.

##### Study group

In the study group, PPV was started with 21% oxygen. After 30 s of effective ventilation, the O_2_ concentration was increased to 100%. CC were initiated only if the lamb went into complete cardiac arrest with HR = 0 bpm.

In both the groups, following asphyxia, resuscitation was initiated by providing PPV using peak inflation pressures (PIPs) of 30–35 cm H_2_O and positive end expiratory pressure (PEEP) of 5 cm H_2_O at a respiratory rate of 40 breaths/min with 21% oxygen using a T-piece resuscitator (Neo-Tee infant, Mercury Medical, Clearwater FL*)* attached to an ETT. The initial pressures of 35/5 cm H_2_O were titrated to deliver standard tidal volumes of 7–9 ml/kg in near-term lambs. We monitored the tidal volume using a Phillips NM3 monitor (Respironics, Andover, MA). At 30 s, in both groups, supplemental oxygen was increased to 100% (this was done to avoid additional variables interfering with the experiment). CC with ventilation at a ratio of 3:1 was initiated, depending on the protocol for the control and study group. The first dose of epinephrine was given at 2 min (min) via the ETT (0.1 mg/kg, 1:10,000 concentrations, and 1 ml/kg) as per NRP recommendations. Subsequent doses of epinephrine starting at 5 min were administered at 3-min intervals through an intravenous line (right jugular venous) until ROSC (0.03 mg/kg, 1:10,000 concentrations, and 0.3 ml/kg). Resuscitation was continued until ROSC or until 20 min from the onset of PPV (whichever was earlier). We defined the start time for ROSC when a sustained HR of ≥100 bpm and DBP ≥ 20 mmHg was attained. Arterial blood gases were drawn and analyzed with ABL 800 FLEX (Brea, CA) at baseline, asphyxia/before resuscitation, every minute during resuscitation, and at ROSC. If ROSC was not achieved, gases were drawn every minute for 20 min. Post ROSC, blood gas was drawn at 1, 2, and 5 min, followed by every 5 min for the first 30 min to measure gas exchange. Subsequent gases were drawn every 15 min for 2 h. Left carotid, left pulmonary, left circumflex coronary artery flows, and invasive blood pressures were continuously monitored to characterize the pulmonary and systemic hemodynamics throughout observation using AcqKnowledge Acquisition and Analysis Software (BIOPAC Systems, Goleta, CA). During the study period, lambs were continuously monitored for pain and discomfort by response to toe pinch and elevation in HR and blood pressure. Intravenous fentanyl (0-2 mcg/kg/hr, titrated to effect) and/or diazepam (0.5 mg/kg bolus as needed) was administered to lambs after ROSC until the study endpoint, with similar doses to neonates if needed.

#### Data collection

Blood flow data was collected continuously. Although only one lamb received CC in the study group, the data is represented as an average and standard deviation based on the events, as the times were variable (Table 2).

#### Sample size estimation

There are no previous studies to evaluate the effect of CC on a neonatal bradycardia model. Based on our preliminary data, we needed at least six lambs in each group to detect an average time difference of 1 min to achieve ROSC with a standard deviation of 0.5 min, with a *p*-value of <0.05 and a power of 0.90. We designed the study based on ILCOR and NRP recommendations and analyzed the difference in outcome.

#### Statistical analysis

The Kolmogorov–Smirnov test was used to determine the parametric/non-parametric distribution of the data, and the analysis was done accordingly. Demographic data are presented as frequencies, percentages, averages/standard deviations, or median/interquartile ranges. The Chi-square test, unpaired *t*-test, analysis of variance (ANOVA), Bonferroni post-hoc test, and non-parametric tests were used as needed. SPSS 29 and SAS 9.4 were used for statistical analysis.

## Results

This is the first asphyxia-induced transitional model of bradycardia. Thirty lambs (*n* = 15 in each group) were instrumented. Lambs with PEA, arrhythmia, or loss of forward flow in the coronary, carotid, and pulmonary circulation after instrumentation but prior to the commencement of the experiment were excluded to ensure the integrity of the methodology of the study and to avoid confounding variables, which is a common challenge in neonatal resuscitation models. After these exclusions, six lambs remained in each control and study group. The exclusion criteria were applied to both groups to maintain homogeneity and validity of the experimental model. The characteristics of the randomized lambs are shown in Table 1.

**Table 1:** Characteristics of bradycardic ovine model.

Parameter	Control (PPV and CC) (*N* = 6)	Study (PPV alone) (*N* = 6)
Gestational age (days)	139 ± 0.4	139 ± 0.5
Birth weight (kg)	3.69 ± 0.4	3.38 ± 0.57
Sex (*N*)	M = 3, F = 3	M = 6
Multiplicity	Singleton = 1 Twins = 5	Singleton = 1 Twins = 3 Triplets = 1 Quadruplet = 1
HR at resuscitation (bpm)	57 ± 9	59 ± 6
pH	7.24 ± 0.05	7.27 ± 0.05
PaCO_2_ (mmHg)	61 ± 8	60 ± 12
PaO_2_ (mmHg)	18 ± 1	23 ± 3

Data represented as average and standard deviation.

In the control group, 2 out of 6 lambs received ETT epinephrine, and 1 out of 6 received both ETT and UV epinephrine. All lambs in the control group received CC. In the study group, only 1 out of 6 lambs received ETT epinephrine.

Following asphyxiation by cord occlusion, the HR at the beginning of resuscitation was 57 ± 9 bpm (Control) vs. 59 ± 6 bpm (Study) (*p* = 0.66). The arterial pH, partial pressure of carbon dioxide in arterial blood (PaCO_2_), and partial pressure of oxygen in arterial blood (PaO_2_) at baseline were not different between the groups, as shown in Table 1.

### Primary outcome

Although not statistically different, the time taken to achieve ROSC was shorter in the study group compared to the control (3 ± 2 min vs. 1.6 ± 1 min, *p* = 0.17). Individual data is shown in Table 2. The incidence of requiring CC was significantly lower in the study group compared to the control group. Only 1/6 lambs (16.7%) required CC in the study group. In the study group, the 1 lamb that received CC went into arrest after 1 min.

**Table 2:** Primary outcomes in the control and study group.

Parameter	Control (PPV and CC) (*N* = 6)	Study (PPV alone) (*N* = 6)
Time to ROSC (min)	3.0 ± 2	1.6 ± 1
Chest compressions *N* (%)	6/6 (100%)	1/6 * (16.7%)

Data represented N (%); average and standard deviation.

*ROSC* return of spontaneous circulation.

*p < 0.05 by chi-square.

### Gas exchange

**Fig. 2:**
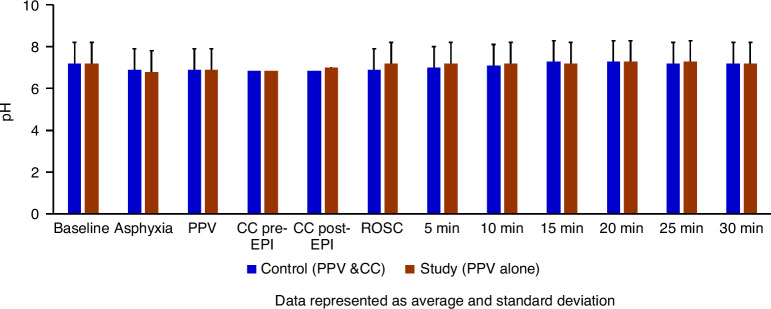
The bar graph shows the arterial pH levels in the control group (blue) and the study group (brown) during resuscitation. There was no difference in pH levels between the control and study groups at fetal baseline, during asphyxia, during resuscitation, or after ROSC. Data are presented as mean and standard deviation. PPV positive pressure ventilation, CC chest compression, EPI epinephrine, ROSC return of spontaneous circulation.

**Fig. 3:**
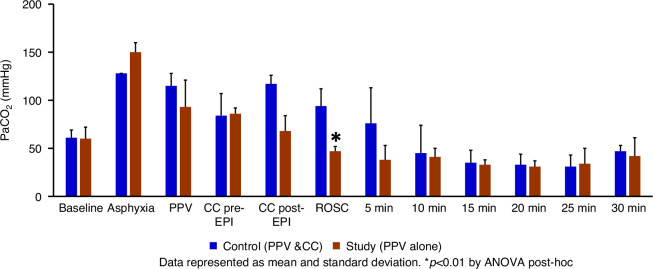
The bar graph shows the preductal arterial pressure of carbon dioxide (PaCO_2_ in mmHg) levels in the control group (blue) and the study group (brown) during resuscitation. There was a significant difference (*p < 0.01) in PaCO₂ level at ROSC between the control group (PPV and CC) and the study group (PPV alone), as determined by ANOVA post-hoc analysis. Data are presented as mean and standard deviation. PPV positive pressure ventilation, CC chest compression, EPI epinephrine, ROSC return of spontaneous circulation.

**Fig. 4:**
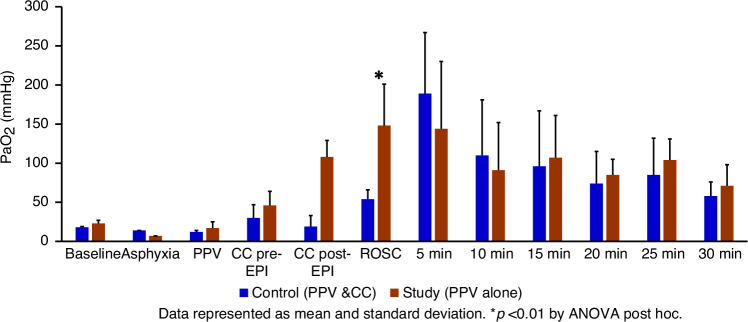
The bar graph shows the preductal arterial pressure of oxygen (PaO_2_ in mmHg) levels in the control group (blue) and the study group (brown) during resuscitation. The study group had significantly higher arterial PaO_2_ levels at ROSC (*p < 0.01), as determined by ANOVA post-hoc analysis. Data are presented as mean and standard deviation. PPV positive pressure ventilation, CC chest compression, EPI epinephrine, ROSC return of spontaneous circulation.

The exhaled tidal volume (TV) in the control group was 7.82 ± 0.4 ml/kg and 7.68 ± 0.7 ml/kg (*p* = 0.7) in the study group. The arterial pH was not different between the control and the study group (Fig. 2). At ROSC, the PaCO_2_ was significantly lower in the study group (47 ± 5 mmHg vs. 94 ± 18 mmHg, *p* < 0.01) (Fig. 3), and PaO_2_ was significantly higher in the study group (148 ± 53 mmHg vs. 54 ± 12 mmHg, *p* < 0.01) (Fig. 4), demonstrating improved gas exchange.

### Pulmonary and systemic hemodynamics

**Fig. 5:**
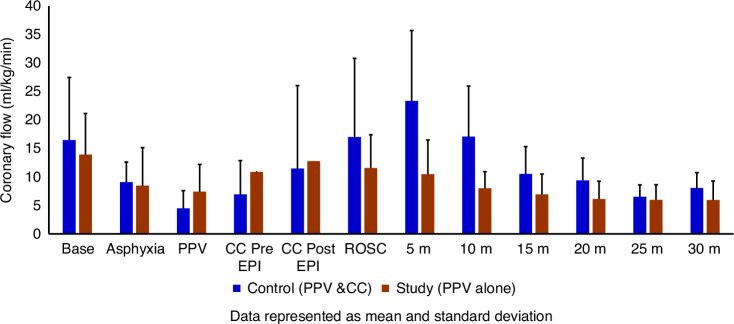
The bar graph shows the peak coronary blood flow (ml/kg/min) in the control group (blue) and the study group (brown) during resuscitation. Peak coronary blood flow was not different in the control and study groups at fetal baseline, during asphyxia, during resuscitation, or after ROSC. Data are presented as mean and standard deviation. PPV positive pressure ventilation, CC chest compression, EPI epinephrine, ROSC return of spontaneous circulation.

**Fig. 6:**
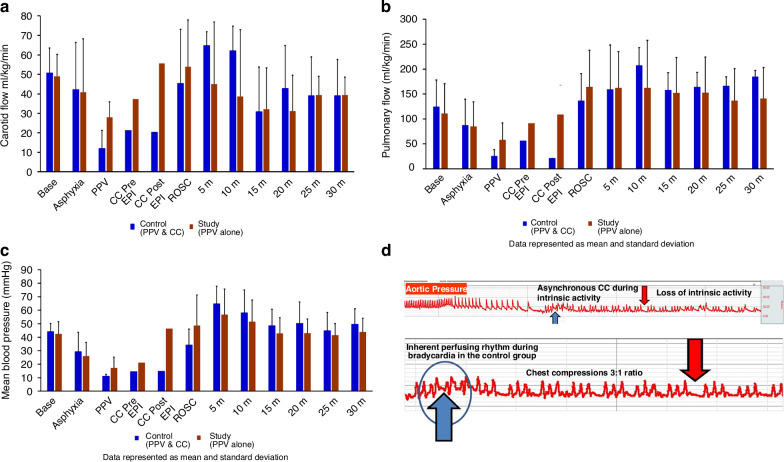
Shows the hemodynamics during the events. It also shows the loss of intrinsic activity during chest compression. **a** The bar graph shows the peak carotid blood flow (ml/kg/min) in the control group (blue) and the study group (brown) during resuscitation. Peak carotid blood flow was not different in the control and study groups at fetal baseline, during asphyxia, during resuscitation, or after ROSC. Data are presented as mean and standard deviation. PPV positive pressure ventilation, CC chest compression, EPI epinephrine, ROSC return of spontaneous circulation. **b** The bar graph shows the peak pulmonary blood flow (ml/kg/min) in the control group (blue) and the study group (brown) during resuscitation. There was no difference in the peak pulmonary blood flow in the control and study groups at fetal baseline, during asphyxia, during resuscitation, or after ROSC. Data are presented as mean and standard deviation. PPV positive pressure ventilation, CC chest compression, EPI epinephrine, ROSC return of spontaneous circulation. **c** The bar graph shows the mean blood pressure (mmHg) in the control group (blue) and the study group (brown) during resuscitation. The mean blood pressure was not different in the control and study groups at fetal baseline, during asphyxia, during resuscitation, or after ROSC. Data are presented as mean and standard deviation. PPV positive pressure ventilation, CC chest compression, EPI epinephrine, ROSC return of spontaneous circulation, BP blood pressure. **d** The BIOPAC snapshot of aortic pressure is shown here. An interesting and consistent finding during bradycardia was that after the initiation of asynchronous chest compressions (CC), the heart’s inherent perfusing rhythm, indicated by the upward blue arrows, was lost, as shown by the downward red arrow, before achieving the return of spontaneous circulation (ROSC).

Coronary blood flow (Fig. 5), carotid blood flow (Fig. 6), and pulmonary blood flow were not different between the groups during chest compressions.

### Loss of inherent heart rate

One of the interesting findings from the BIOPAC snapshot of blood pressure and blood flow recordings post-resuscitation was the loss of the inherent perfusing rhythm of the heart, as shown in Fig. 6d. The inherent perfusing rhythm was lost during external CC, which was not coordinated with the inherent cardiac activity. Providing asynchronous CC may not support the existing cardiac rhythm. Loss of perfusing rhythm during chest compressions was continuously monitored using the BIOPAC system, which provided real-time data from the EKG, arterial blood pressure, and blood flow measurements. An example of inherent HR is shown in Fig. 6d, which was evaluated retrospectively from BIOPAC tracings. These parameters were actively analyzed during chest compressions to detect any loss of perfusion. This occurred with 5/6 lambs in the control group, and the time for this loss of inherent rhythm post external asynchronous CC was 80 ± 40 s.

## Discussion

With growing evidence from translational research, NRP can now reevaluate historically consensus-based protocols.^2,6,11^ The rationale for performing CC in neonates with bradycardia has traditionally been more consensus-based than evidence-driven. Bradycardia in the newborn is often the result of inadequate lung inflation or profound hypoxemia.^1^ Establishing adequate ventilation is the most effective way to treat bradycardia. To date, research on CC in neonates, either with bradycardia or total arrest, has focused on different techniques, the depth of CC, use of sustained inflation, various oxygen concentrations, and assisted devices to assess effective CC and ROSC.^12,13^ To our knowledge, this is one of the first studies to evaluate the effect of CC in a bradycardic transitioning ovine model, and was designed to examine the current clinical practice. The fact that all lambs in the control group required CC allowed for a comparative evaluation of the intervention’s efficacy between the groups. This outcome confirms the importance of providing effective PPV and raises concerns about how external CC may impact inherent cardiac function.

In neonates, inherent HR is a significant determinant of cardiac output compared to adults. Previous studies have found that end-diastolic volume is essential in cardiac fetal production, as Frank Starling’s relationship exists in the fetal heart.^14–17^ Ventricular filling and myocardial perfusion occur during the peak phase of diastole, and cerebral perfusion occurs during the peak phase of systole. In most newborns experiencing bradycardia secondary to birth asphyxia, which is the most common cause of neonatal resuscitation, the heart is structurally healthy, but the myocardium is ischemic and acidotic. External CC that are asynchronous during bradycardia, i.e., not synchronized to the inherent rhythm of the heart, could interfere with the natural systolic and diastolic phases, which in turn may reduce the time for ventricular filling, affecting the preload.^6^ A piglet study did not find any difference in cardiac output with asynchronous CC at 90 CC per minute, however these piglets had their PDA ligated.^18^ Performing external CC asynchronously (90 CC–30 breaths) when the HR is <60 bpm could lead to impaired ventricular filling, resulting in peak systolic cerebral blood flows being reduced. The presence of a patent ductus further complicates this, with bidirectional blood flows potentially decreasing coronary flows. This raises questions about whether evidence-based physiologic data support the current consensus-based practice. The occurrence of PEA or cardiac arrest prior to the start of the experimental protocol in a subset of lambs likely stemmed from the severe asphyxia induced by cord clamping, a common challenge in studies involving neonatal resuscitation models. The goal of this study was to study bradycardia with functioning blood flow, which is currently not possible to evaluate clinically in a prospective randomized design.

Inherent higher HR is crucial for newborns, but does this physiological characteristic apply when external CC are used for bradycardia? In adults with asystole, external CC generates forward blood flow according to the “cardiac” and “thoracic” pump theories.^18^ However, this may not be the case for bradycardia, particularly when performing external CC at one-third of the chest depth, especially if there is inadequate ventricular filling.

The use of EKG has increased the detection of HR in the delivery room and has also increased the incidence of CC.^2^ However, the survival rate of neonates has remained the same before and after the introduction of EKG in the delivery room.^4^ Focusing on providing CC pulls the resuscitator’s attention from the most important step in neonatal resuscitation: providing adequate ventilation of a liquid-filled newborn lung. Halling et al., in their registry study involving 1153 neonates, have shown that CC was initiated within the first minute of life in 76% of the events and before endotracheal intubation in 79% of the events.^19^ NRP recommendation for 30 s of effective ventilation, with endotracheal intubation, is consensus-based and not evidence-based.

Our data suggests that focusing on ventilation rather than CC during bradycardia with an existing inherent HR may improve gas exchange and decrease the need for CC. The PaCO_2_ and PaO_2_ levels improved significantly in the study group compared to the control group, suggesting that CC could interfere with gas exchange. Since most of the newborns are depressed secondary to birth asphyxia, the focus of neonatal resuscitation should be on ventilation as recommended by NRP.^1^ Although we did not find a difference in hemodynamics during CC between the groups, we had only one lamb that required CC in the study group, so statistical comparison was not possible. It is important to note that all lambs in control group required CC per standard NRP recommendations, while only one animal in the study group did not respond to ventilation alone. The minimal inherent HR in this bradycardic lamb was sufficient to improve gas exchange, causing a reduced time to ROSC. When there is no inherent HR, providing effective ventilation is futile without circulation.^18^

Another important finding we have reported previously and observed in our study is the loss of inherent cardiac activity while performing CC in a bradycardia model.^6^ Previously, in a pediatric population, Khera et al. reported higher mortality in intensive care unit patients when CC was initiated for bradycardia with a pulse, which subsequently progressed to pulselessness.^8^ The underlying conditions of these patients remain unclear, but we speculate based on our findings, that the asynchronous nature of CC while the heart is trying to compensate during bradycardia could interfere with ROSC. Unless proven, we can never assume that a few minutes of cardiac compressions will not affect long-term cardiac outcomes and exercise tolerance. In the presence of asphyxia and acidosis, which could impact the myocardium and render it unhealthy, extensive resuscitation can cause hypoxic injury, and the effect of whole-body hypothermia can lead to morbidities such as pulmonary hypertension and additional strain on the heart. Hence, more clinical data from the neonatal population who underwent resuscitation are needed to understand the effect of CC during bradycardia and the loss of perfusing rhythm on their long-term cardiac outcome and exercise tolerance.

Our study has limitations. Lambs are a different species with a chest shape that differs from human neonates. However, they have been used in neonatal research because their lung development resembles human neonates. Our focus was to simulate the newborn transition at birth during a bradycardic episode. The study randomization was selected before resuscitation by design and was not blinded. All lambs were delivered by C-section. Selection criteria included lambs with persistent stable bradycardia, measuring effective forward blood flow at the start of ventilation with no arrhythmia or PEA. We also did not perform bag-and-mask ventilation prior to initiating PPV via ETT and chest compressions. The absence of corrective ventilation steps such as mask adjustments, reposition of head and neck, suction of mouth and nose, open airway, pressure increase, alternate airway (MR SOPA) as recommended by NRP may influence the results. To minimize confounding factors, all lambs were intubated before asphyxia was induced, and the entire resuscitation process was conducted in a controlled environment. A key limitation of this study is the open-chest model required for placement of flow probes, which may alter hemodynamic responses to chest compressions compared to an intact chest. However, the instrumentation was similar in both control and study groups and was not influenced by assignment bias. The coronary flow probe was essential for obtaining data evaluating myocardial perfusion with a direct measure of coronary blood flow, which is a novel aspect of our research. A randomized study like this in humans would not be ethically feasible. Finally, we recognize that this model primarily focuses on asphyxia-induced bradycardia during neonatal resuscitation in the delivery room, which may limit the broader applicability of the findings to other causes of neonatal arrest.

Our significant strengths include the use of the first ever asphyxia induced bradycardia model and the measurement of blood flow in the left circumflex artery as a surrogate for coronary flow instead of using coronary perfusion pressure. This is also the first scientific study to evaluate the current recommendations for initiating CC in a bradycardic newborn. We aim to investigate the effects of lowering the heart rate threshold for initiating CC during neonatal resuscitation.

## Conclusion

Focusing on providing effective PPV reduced the need for CC, improved gas exchange at ROSC, and preserved inherent flows in a near-term ovine model of bradycardia compared to current NRP resuscitation guidelines.

Diverting attention to providing CC may distract neonatal resuscitation providers from focusing on effective PPV. The current recommendations from NRP remain the standard of care until further evidence becomes available. Registry studies/future clinical studies, along with follow-up, are needed to understand the indication for CC.

## Supplementary information

Supplementary information

Supplementary information

## Data Availability

The data will be available upon reasonable request after completion with MRI findings of the brain.
