# Supplementary material for: Neonatal resuscitation for bradycardia (HR < 60 bpm)—an alternate approach using an ovine model
Source: Pediatr Res. 2025 Oct 7;99(5):1992–8. doi: 10.1038/s41390-025-04444-9 (PMC13221291; doi:10.1038/s41390-025-04444-9)
Supplement: Supplementary file 1 — Supplementary information [file 41390_2025_4444_MOESM1_ESM.pdf]

Supplemental file:

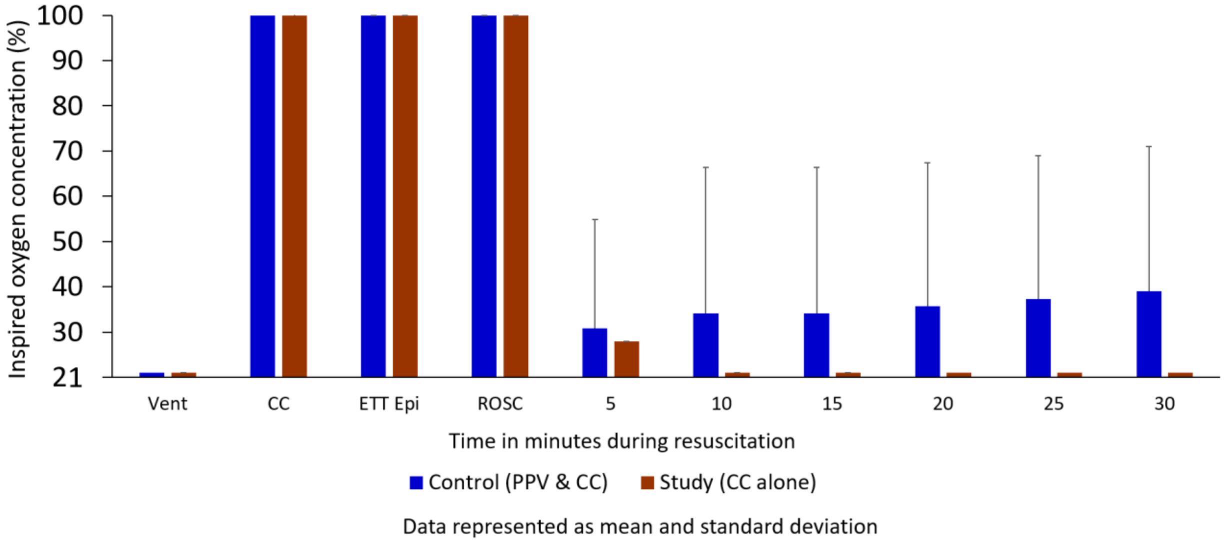

Use of supplemental oxygen:

The bar graph shows the inspired oxygen concentration (%) in the control group (blue) and the study group (brown) during resuscitation. There was no difference in the inspired oxygen concentration used between the control and study groups at the onset of ventilation, during resuscitation, or after ROSC. Data are presented as mean and standard deviation.

PPV – positive pressure ventilation, CC – chest compression, EPI – epinephrine, ROSC- return of spontaneous circulation

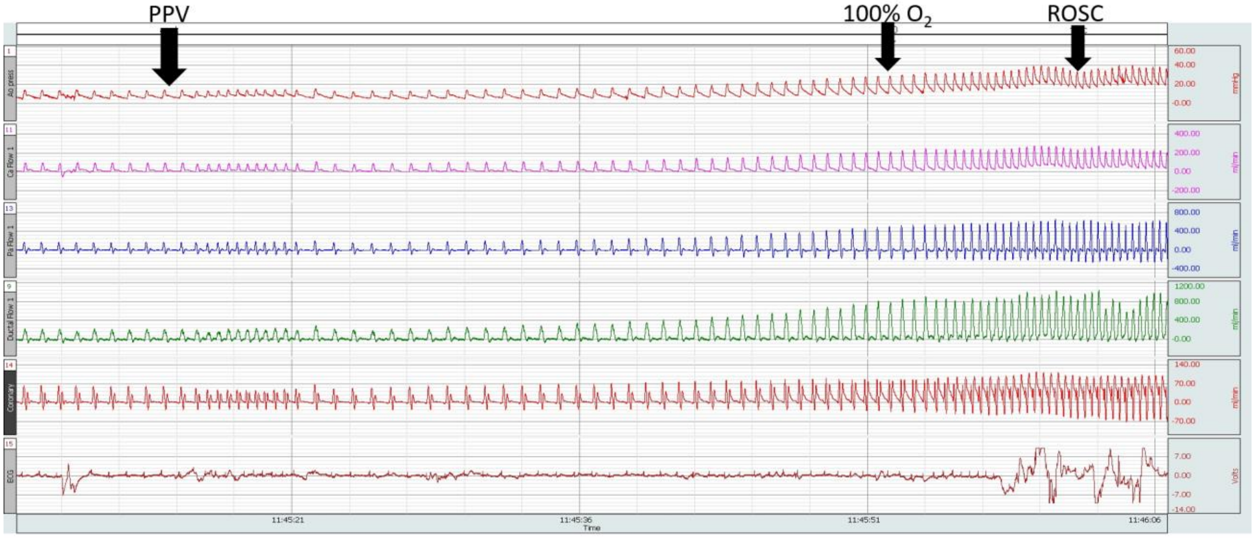

BIOPAC snapshot shows the start of ventilation (PPV), increase to 100% supplemental oxygen and ROSC (return of spontaneous circulation) in a bradycardic lamb model.
